# Genetically Confirmed Malignant Hyperthermia in a Six-Week-Old Infant: A Case Report

**DOI:** 10.7759/cureus.27010

**Published:** 2022-07-19

**Authors:** Christopher M Edwards, Thomas K Jenkins, Nikolaus Gravenstein, Amy M Gunnett, Timothy W Martin

**Affiliations:** 1 Anesthesiology, University of Florida College of Medicine, Gainesville, USA

**Keywords:** congential diaphragmatic hernia, genetic testing, dantrolene, pediatrics, malignant hyperthermia

## Abstract

A six-week-old 3.9-kg infant presented for microlaryngoscopy and diaphragmatic hernia repair. While positioning for laparoscopy after microlaryngoscopy, the infant developed muscle rigidity, worsening hypercarbia, tachycardia, and early hyperthermia. Sevoflurane was discontinued, and the clinical picture indicating malignant hyperthermia (MH) resolved without dantrolene. Subsequent genetic testing revealed that both the patient and his father carried a mutation in the *RYR1* gene that is diagnostic for MH. This is the second youngest genetically confirmed case of MH. This case adds to a limited body of evidence regarding MH presentation and diagnosis in neonates and infants.

## Introduction

Malignant hyperthermia (MH) is a severe hypermetabolic reaction of genetically abnormal skeletal muscle, triggered by exposure to volatile anesthetics and/or the depolarizing muscle relaxant succinylcholine. If it is not promptly recognized and treated, the clinical effects of MH may rapidly progress to rhabdomyolysis, hyperkalemia, multiorgan system failure, and death. MH mortality still approaches 10% [1] despite a thorough understanding of the pathophysiology and widespread availability of the effective antidote dantrolene.

Although the prevalence of MH-causative genetic mutations is as high as 1 in 2750, the actual incidence of clinical MH is much lower [2]. Reports of MH in children younger than two years of age are rare [3]. It is proposed that MH may present differently in neonates and infants due to decreased skeletal muscle mass, but few case reports exist to examine this hypothesis. We report what we believe is the second youngest genetically confirmed case of MH [4] and the youngest that was resolved without dantrolene.

Written informed consent and written Health Insurance Portability and Accountability Act (HIPAA) authorization were obtained from the parents to report this case, and this article adheres to the applicable Enhancing the Quality and Transparency of Health Research (EQUATOR) guideline.

## Case presentation

A six-week-old, 3.9-kg male infant presented to the operating room for microlaryngoscopy and laparoscopic diaphragmatic hernia repair. This infant had been transferred to the neonatal intensive care unit (NICU) from another institution because of failure to wean from continuous positive airway pressure. In addition to retrognathia, he was found to have a ventricular septal defect and was suspected to have a central congenital diaphragmatic hernia (CDH).

**Figure 1:**
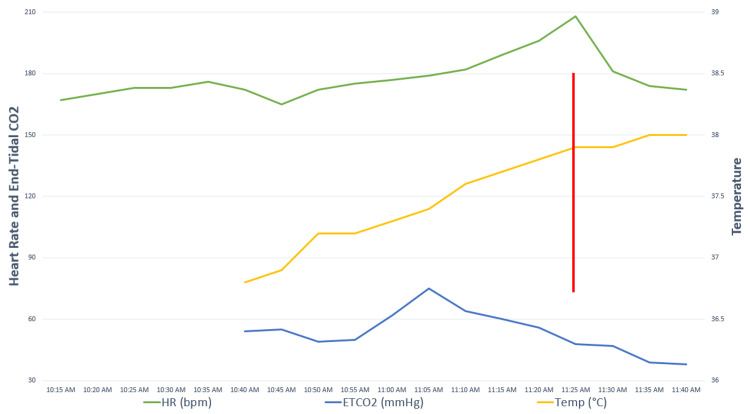
Intraoperative vital signs. Concern for malignant hyperthermia developed during the period from 10:50-11:10 am, following microlaryngoscopy and supraglottoplasty and while repositioning the infant with the intent to proceed with diaphragmatic hernia repair. The red bar shows the approximate time of femoral arterial blood gas analysis (Table 1). Heart rate (beats per minute) and end-tidal carbon dioxide (mmHg) over time are displayed on the left y-axis. Temperature (degrees Celcius) over time is displayed on the right y-axis.

Intraoperative vital signs and blood gas data are presented in Figure 1 and Table 1. Initially, a combination of intravenous ketamine and inhaled sevoflurane via a face mask was used to anesthetize the infant for microlaryngoscopy. Pediatric otolaryngology found that the infant had redundant arytenoid tissue and performed a supraglottoplasty, then placed a 3.5-cuffed endotracheal tube (ETT). We continued to deliver maintenance sevoflurane via ETT, and the infant was repositioned from approximately 10:50 to 11:10 AM with the intention of proceeding with laparoscopy and CDH repair.

**Table 1:** Femoral arterial blood gas results.

Intraoperative blood gas	
Sodium	135
Potassium	4.8
Calcium (ionized)	1.28
Glucose	141
Hematocrit	33
Lactic acid	1.5
pH	7.34
pCO_2_	45.0
pO_2_	471
Bicarbonate	24.3
Base excess	−2

However, during repositioning, the infant developed progressive hypercarbia and tachycardia. His end-tidal carbon dioxide (ETCO_2_) increased from 50 mm Hg to a peak of 75 despite vigorous manual ventilation. His heart rate increased from 170 beats per minute to a peak of 209. Concurrently, his rectal temperature increased from 36.8 °C to a peak of 38. The infant developed generalized muscle rigidity, which made positioning difficult, with his arms flexed over his chest and his legs flexed over his pelvis. This rigidity persisted despite administering 5 mg of intravenous rocuronium. The team became concerned about MH; therefore, we discontinued inhalational anesthetics and called for the MH cart and help.

During the next 15 min, the infant’s muscle tone relaxed, his ETCO_2_ and heart rate returned to baseline, and a femoral arterial blood gas depicted insignificant respiratory or lactic acidosis with mild hyperkalemia (Table 1). Given these reassuring results and trends, the team decided not to administer dantrolene. Laparoscopy and CDH repair were not initiated, and the infant was returned intubated to the NICU. His serum creatine kinase (CK) was elevated at 1056 U/L on arrival at the NICU and peaked at 4914 the next morning. No recurrence of MH was noted. The infant was subsequently returned to the operating room for CDH repair with an uneventful, non-triggering anesthetic. This total intravenous anesthetic consisted primarily of fentanyl, ketamine, and midazolam.

Given the concern for MH susceptibility, genetics was consulted, and whole exome sequencing was performed. Two variants of the RYR1 gene were identified: c.6487C>T and c.5011G>A. c.6487C>T is considered diagnostic for MH [5] and is shared with the father, while c.5011G>A is considered a variant of uncertain significance and is shared with the mother. Genetic testing of two older siblings is in progress, and the family has been counseled regarding the need for non-triggering anesthetics.

## Discussion

This case adds to a limited body of evidence regarding the presentation of MH in neonates and infants. Tsutsumi et al. reported a six-day-old neonate who experienced an MH crisis [4], also during attempted CDH repair, which resolved with 1 mg/kg of dantrolene. The peak CK value, in that case, was 3795 U/L, similar to our patient and lower than the peak CK values typically seen in MH [6]. In fact, a 2014 analysis of North American Malignant Hyperthermia Registry (NAMHR) reports by Nelson and Litman found that children aged 0 to 24 months old had lower peak CK values after an episode of MH than older children [3]. They hypothesized that this is due to greater muscle mass in older children. They also found that the maximum temperature during an MH episode was higher in children aged 13 to 18 years (40.2 °C) than in children aged 0 to 24 months or 2 to 12 years (38.4 °C and 38.2 °C, respectively), again consistent with the modest degree of hyperthermia observed in our case.

Both our case and that reported by Tsutsumi et al. [4] highlight the potential challenges in diagnosing MH in neonates, especially in those with respiratory comorbidities such as CDH. These patients may have baseline tachycardia and hypercarbia, making the appearance of these frequent signs of MH less alarming. The patient in the case described by Tsutsumi was diagnosed with MH and successfully treated with dantrolene, but only after the patient’s temperature exceeded 39 °C and ETCO_2_ exceeded 100 mm Hg. In our case, although MH was considered and triggering anesthetics were discontinued, subsequent normalization of physiologic parameters and an unremarkable blood gas led to doubt in the diagnosis, and dantrolene was not administered. It is important to maintain a high degree of suspicion for MH in neonates even if the blood gas and CK values are not as abnormal as expected. Particular attention should be paid to unexpected muscular rigidity, which was present in both cases. The lack of a muscle relaxing response to rocuronium is also a hallmark of MH because MH is not operational at the neuromuscular junction; it is a direct muscle process.

Although our case was resolved without dantrolene, and the case described by Tsutsumi was resolved with a smaller than usual bolus dose of dantrolene [4], dantrolene should be administered at the full initial bolus dose of 2.5 mg/kg if MH is suspected. Additional dantrolene can be administered as needed until signs and symptoms of MH abate. Nelson and Litman found no evidence of increased side effects from dantrolene in the youngest cohort [3]. Rather, they found only an 8.8% incidence of muscle weakness after dantrolene administration in patients aged 0 to 24 months, compared to a 24.2% incidence of weakness in patients aged 13 to 18 years. Further research may be warranted to determine whether MH aborts more easily in neonates because of decreased skeletal muscle mass.

Finally, our case illustrates the growing role of genetic testing for MH susceptibility. Although in vitro contracture testing remains the most sensitive way to investigate MH susceptibility, most centers do not perform this test in patients younger than five years of age due to the amount of quadriceps muscle that is harvested [7,8]. Whole exome sequencing can be performed at any age and, if positive for a diagnostic MH mutation, establishes MH susceptibility and eliminates the need for invasive contracture testing. Furthermore, genetic testing can be performed at the family level, is cost-effective, and can quickly establish MH susceptibility in family members who share the same pathogenic genetic variant as the index patient [2,9].

## Conclusions

MH is a rare but severe complication of exposure to volatile anesthetics. Our case, one of the youngest genetically confirmed cases of MH, adds to limited evidence that this process may present differently in neonates and infants due to decreased skeletal muscle mass. Thus, it is important to maintain a high degree of suspicion for MH in infants, even if blood gas and CK values are not as abnormal as would be expected in older patients. While we were fortunate that MH resolved simply with discontinuation of the triggering inhalational anesthetic, intravenous dantrolene should be administered whenever an MH crisis is suspected, as there is no evidence of increased dantrolene side effects in young patients. Finally, this case sheds light on the important role of genetic testing for MH susceptibility, especially in patients too young for muscle contracture testing. Whole exome sequencing quickly established MH susceptibility in our patient as well as the father, who shared a known pathogenic variant.
